# Ethyl Acetate Fraction from *Eleutherococcus divaricatus* Root Extract as a Promising Source of Compounds with Anti-Hyaluronidase, Anti-Tyrosinase, and Antioxidant Activity but Not Anti-Melanoma Activity

**DOI:** 10.3390/molecules29153640

**Published:** 2024-08-01

**Authors:** Jakub Gębalski, Milena Małkowska, Sylwia Wnorowska, Dorota Gawenda-Kempczyńska, Maciej Strzemski, Magdalena Wójciak, Artur Słomka, Jan Styczyński, Daniel Załuski

**Affiliations:** 1 Department of Pharmaceutical Botany and Pharmacognosy, Ludwik Rydygier Collegium Medicum, Nicolaus Copernicus University, 85-094 Bydgoszcz, Poland; milena.malkowska@cm.umk.pl (M.M.); dgawenda@cm.umk.pl (D.G.-K.); daniel.zaluski@cm.umk.pl (D.Z.); 2 Department of Medical Chemistry, Medical University of Lublin, 20-093 Lublin, Poland; sylwiaw@gmail.com; 3 Department of Analytical Chemistry, Medical University of Lublin, 20-093 Lublin, Poland; maciej.strzemski@umlub.pl (M.S.); magdalena.wojciak@umlub.pl (M.W.); 4 Department of Pathophysiology, Ludwik Rydygier Collegium Medicum, Nicolaus Copernicus University, 85-094 Bydgoszcz, Poland; artur.slomka@cm.umk.pl; 5 Department of Pediatric Hematology and Oncology, Ludwik Rydygier Collegium Medicum, Nicolaus Copernicus University, 85-094 Bydgoszcz, Poland; jstyczynski@cm.umk.pl

**Keywords:** *Eleutherococcus divaricatus*, human hyaluronidase, human tyrosinase, metabolites

## Abstract

*Eleutherococcus divaricatus* (Siebold and Zucc.) S. Y. Hu. has been used in Traditional Chinese Medicine (TCM) due to its anticancer, immunostimulant, and anti-inflammatory activities. However, its mechanism of action and chemical composition are still insufficiently understood and require more advanced research, especially for cases in which anti-inflammatory properties are beneficial. The aim of this study was to evaluate the impact of *E. divaricatus* root extracts and fractions on proinflammatory serum hyaluronidase and tyrosinase in children diagnosed with acute lymphoblastic leukemia. Antioxidant and anti-melanoma activities were also examined and correlated with metabolomic data. For the first time, we discovered that the ethyl acetate fraction significantly inhibits hyaluronidase activity, with mean group values of 55.82% and 63.8% for aescin used as a control. However, interestingly, the fraction showed no activity against human tyrosinase, and in A375 melanoma cells treated with a doxorubicin fraction, doxorubicin activity decreased. This fraction exhibited the most potent antioxidant activity, which can be attributed to high contents of polyphenols, especially caffeic acid (24 mg/g). The findings suggest an important role of the ethyl acetate fraction in hyaluronidase inhibition, which may additionally indicate its anti-inflammatory property. The results suggest that this fraction can be used in inflammatory-related diseases, although with precautions in cases of patients undergoing chemotherapy.

## 1. Introduction

Enzymes such as hyaluronidase and tyrosinase, which are naturally present in the human body, play crucial roles in a variety of physiological processes [1,2,3,4,5,6]. This includes the facilitation of fertilization and the production of melanin. However, these enzymes are also implicated in several pathogenic processes, including the formation of cancerous metastases and the development of age spots [7]. The discovery and subsequent characterization of inhibitors of hyaluronidase could potentially pave the way for the creation of novel anticancer treatments, effective contraceptives, and antidotes for various venoms and toxins [8,9,10,11,12]. Conversely, inhibitors of tyrosinase have potential applications in the cosmetic and pharmaceutical industries as skin-lightening agents or in the treatment of pigmentation-related skin conditions [13,14,15,16,17].

The Araliaceae family encompasses trees, shrubs, and climbers. This family boasts numerous species that are widely utilized as ornamental plants, including *Schefflera arboricola* L. and *Fatsia japonica* (Thunb.) Decne. & Planch [18]. Notably, the Araliaceae family also comprises plants of significant medicinal value such as ginseng (*Panax* spp.) and ivy (*Hedera* spp.) [19,20,21]. The representative of this family is also *Eleutherococcus divaricatus* (Siebold and Zucc.) S. Y. Hu., a plant that has been used in Far Eastern traditions for centuries [22].

*E. divaricatus* root contains many compounds, including eleutherosides, flavonoids, triterpenoids, and phenolic acids. The main metabolites vary considerably and are called the eleutherosides, with eleutherosides B (syringin 4-*β*-D-glucoside) and E ((−)-siringaresinol 4,4″-*O*-*β*-D-diglucoside) accounting for the majority. *E. divaricatus* is a plant that has been used for medicinal purposes in traditional medicine systems for many years. It has various pharmacological properties, such as anti-inflammatory, anti-cancer, anti-depressant, antidiabetic, anti-fatigue, neuroprotective, hepatoprotective and immunostimulative activities [23,24,25,26,27,28]. Despite its long history of use in ethnopharmacology to treat the above-mentioned diseases, the mechanism of its action remains largely unexplored. Załuski’s et al. previous research indicated the presence of MMP-1 and MMP-9 inhibitors in chloroform extracts [29]. 

We hypothesized that the roots of *E. divaricatus* contain compounds with anti-hyaluronidase (an enzyme related to tissue degradation) and anti-tyrosinase (an enzyme involved in melanin production) activity. To prove our hypothesis, HPLC-PDA, UHPLC-DAD/ESI-TOF-MS, and biological techniques were used. To obtain more reliable results, we studied both commercially available enzymes (hyaluronidase and tyrosinase), as well as serum hyaluronidase from children diagnosed with acute lymphoblastic leukemia. Additionally, antioxidant and anti-melanoma activities were tested. 

## 2. Results and Discussion

### 2.1. Chemical Panel

Phytochemicals, which are very often characteristic of only a small group of plants, are responsible for their pharmacological effects. The *Eleutherococcus* genus is rich in large numbers of compounds, which makes it is a potential source of plant-based medicines. However, the activity of traditional medicines is usually caused by the combination of compounds, which means that no single active compound can be isolated. In some cases, a fractionation process is the most reasonable approach, which leads to the attainment of active fractions. The first step of this study was to determine the most effective solvent for extraction, expressed as the lowest IC_50_ value for enzyme inhibition. On the basis of these results, 75% methanol extract was chosen for phytochemical analysis as a promising source of inhibitors of hyaluronidase and tyrosinase. Subsequently, using liquid–liquid extraction, 75% methanol extract was fractionated for four fractions. Fractionation resulted in 2.02, 1.02, 5.11, and 9.26 g of n-hexane, ethyl acetate, n-butanol, and water mass fraction, respectively. 

The total contents of phenolic compounds (TPC), flavonoids (TFC), and phenolic acids (TPAC) are shown in Table 1. Our study revealed that the ethyl acetate fraction had the highest concentrations of polyphenols (110.89 ± 6.32 mg/g), flavonoids (27.95 ± 4.11 mg/g), and phenolic acids (2.81 ± 0.48 mg/g). Literature data do not provide much information about polyphenols in *Eleutherococcus* spp., especially with respect to fractions. Załuski et al. studied ethanolic extract obtained from the roots of *E. divaricatus*, which contained 6.9 ± 0.4 mg GAE/g polyphenols per dry sample [30]. In turn, Adamczyk et al. reported that the polyphenol and flavonoid contents in 75% MeOH extracts were equal 9.4 ± 0.9 gGAE/g and 6.5 ± 1.1 gQE/g, respectively [31]. The contents of polyphenols, flavonoids, and phenolic acids of hydrophobic–hydrophilic extract from the roots of *E. senticosus* enriched with naringenin were 159.27 ± 2.73 mgGAE/g, 137.47 ± 5.23 mgQE/g, and 79.99 ± 3.57 mgCAE/g, respectively [32]. 

**Figure 1:**
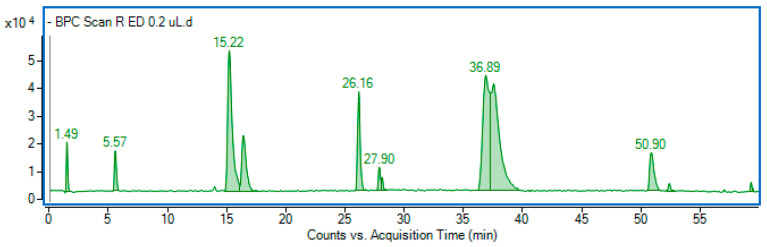
Base peak chromatogram (BPC) of the ethyl acetate fraction obtained in negative ionization mode.

In the next step, the phenolic composition of the fractions was characterized using mass spectrometry. Chromatographic parameters and mass spectra were compared with standards, or components were tentatively identified based on the literature. A representative chromatogram of the most abundant fraction, ethyl acetate, is shown in Figure 1. The mass data used for identification are summarized in Table 2.

**Figure 2:**
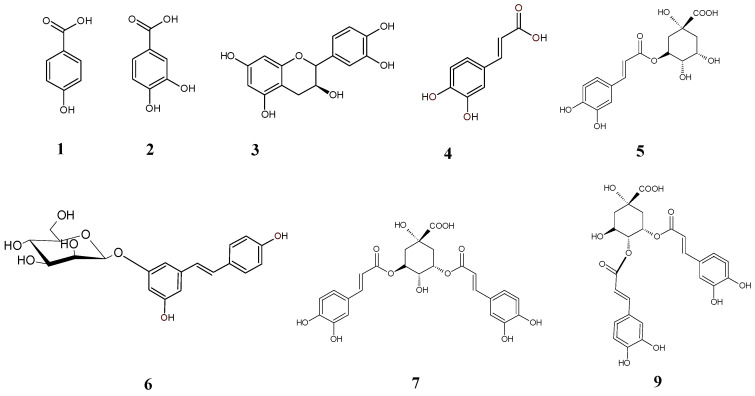
Formulae of the compounds present in the EtOAc fraction according to Table 2.

The analysis revealed the presence of 10 compounds, predominantly derivatives of cinnamic acid such as caffeic acid and its derivatives, including chlorogenic acid, 3,5-dicaffeoylquinic acid, dicaffeoylquinic acid, and 4,5-dicaffeoylquinic acid (Figure 2, Table 3). Furthermore, the fraction contains derivatives of benzoic acid, such as protocatechuic acid, hydroxybenzoic acid, and a diferulic acid derivative, along with a low amount of catechin. None of the investigated eleutherosides (eleutherosides B and E) were found in the fractions. 

The fractions were rich in dicaffeoylquinic acid and chlorogenic acid. It is very interesting that EtOAc was rich in caffeic acid as a representative of simple phenolic acids. A similar phytochemical composition was observed in methanolic extracts obtained from *E. henryi* leaves, which contained caffeoylquinic acid derivatives such as 5-caffeoylquinic acid (5-CQA—27.54 mg/g), 4-caffeoylquinic acid (4-CQA—5.91 mg/g), 3,4-dicaffeoylquinic acid (3,4-DCQA—0.66 mg/g), 3,5-dicaffeoylquinic acid (3,5-DCQA—5.91 mg/g), 1,5-dicaffeoylquinic acid (1,5-DCQA—0.853 mg/g), and 4,5-dicaffeoylquinic acid (4,5-DCQA—3.81 mg/g) [33]. A 75% methanolic extract of *E. divaricatus* was found to contain benzoic acid (salic acid and protocatechuic acid) and cinnamic acid (caffeic acid, ferulic acid, and *p*-coumaric acid) derivatives [31]. Phenolic acids such as trans-4-hydroxycinnamic acid, trans-caffeic acid, and methyl caffeate were found in the methanolic extract from the stem of *E. divaricatus*. In addition, the extract was rich in phenolic alcohols (4-(3-methoxy-1-propen-1-yl)-1,2-benzenediol, coniferyl alcohol, and 4-[(1*E*)-3-methoxy-1-propenyl]phenol) and stilbens ((þ)-pinoresinol, (þ)-medioresinol, (þ)-syringaresinol, acanthoside B, obtusifoside A, acanthoside D, (þ)-sesamin, (þ)-lariciresinol-9-O-*β*-D-glucopyranoside, (þ)-alangilignoside C, and (þ)-salvadoraside) [34]. 

### 2.2. Anti-Enzymatic Panel

#### 2.2.1. Inhibition of Bovine Hyaluronidase (bHYAL) and Fungal Tyrosinase (mTYR) by Crude Extracts and Fractions

The *Eleutherococcus* genus, known for its diverse phytochemical composition, exhibits a wide range of biological activities. The *Eleutherococcus* species have been used in traditional medicine for centuries, offering benefits such as adaptogenic, immunostimulant, stress-combating, anti-fatigue, antioxidant, anti-inflammatory, anti-tumor, neuroprotective, and antidiabetic properties.

Our study explored the impact of *E. divaricatus* root extract on the activity of bovine hyaluronidase (bHYAL) and fungal tyrosinase (mTYR). The initial phase of the study was dedicated to determining the most effective solvent for extraction, expressed as the lowest IC_50_ value for enzyme inhibition (Table 4). The IC_50_ values for bovine hyaluronidase ranged between 100.8 and 181.27 µg/mL, and those for tyrosinase ranged between 103.6 and 274.37 µg/mL. The most active appeared to be 75% methanol, with an IC_50_ of 100.8 µg/mL for bHYAL and 103.6 µg/mL for mTYR. 

In the next step, the 75% methanol extract was subjected into liquid–liquid extraction using nonpolar, medium-polar, and polar solvents (n-hexane, ethyl acetate, n-butanol, and water, respectively). It was found that ethyl acetate fraction showed the highest activity, with an IC_50_ value equal to 27.5 µg/mL for bHYAL and equal to 65.5 µg/mL for mTYR (Table 5). It should be noted that the activity of *E. divaricatus* was stronger than that of the positive control, aescin (IC_50_ = 388.8 ± 1.81 µg/mL). In the case of tyrosinase, none of the fractions showed activity exceeding the value for kojic acid (IC_50_ = 4.44 ± 0.06 µg/mL). The activities of phenolic acids and eleutherosides present in the highest amounts were further determined (Table 6). For both bHYAL and mTYR, the most active compound was caffeic acid (bHYAL—IC_50_ = 111.34 ± 3.59 µg/mL; mTYR—IC_50_ = 60.77 ± 2.37 µg/mL). Eleutherosides B, E, and E1 showed no activity. 

There are many research papers investigating the effects of phenolic acids on tyrosinase and hyaluronidase. In a previous study, the fruits of *E. divaricatus* demonstrated moderate inhibitory activity against hyaluronidase and weak anti-tyrosinase activity (IC_50_ = 0.45 and IC_50_ = 2.67 mg/mL, respectively) [35]. Isolated phenolic acids (derivatives of seric acid) from *Oenanthe javanica* inhibited the activity of bovine hyaluronidase (IC_50_ = 0.19–1.33 mM) [36]. Cimicifugic acids K-N (IC_50_ = 102–255 μM) isolated from the aboveground parts of *Cimicifuga simplex* and *C. japonica*, exhibited more potent hyaluronidase-inhibitory activities than rosmarinic acid (IC_50_ = 545 μM) [37]. 

#### 2.2.2. Inhibition of Human Hyaluronidase (hHYAL) and Human Tyrosinase (hTYR) in Blood Samples from Children Diagnosed with Acute Lymphoblastic Leukemia by Ethyl Acetate Fraction

Hyaluronidase and tyrosinase contribute to the progression of many diseases, which very often have a cancerous background, and their overactivity is observed. Taking this into consideration, we decided to establish the level of these enzymes in blood samples from acute leukemia patients and to examine the influence of the ethyl acetate fraction on their activity. We chose leukemic patients because it is known that in their case, e.g., hyaluronidase levels are high [38]. Five boys with a median age of 4.5 years diagnosed with acute lymphoblastic leukemia (ALL) before starting treatment were included in the study. Serum levels of hHYAL ranged between 25.20 and 162.15 ng/mL, while hTYR levels ranged between 4.68 and 78.94 ng/mL (Table 7). The obtained results show that the ethyl acetate fraction contains inhibitors of hyaluronidase with aescin-like activity (Table 7). The EtOAc fraction inhibited hHYAL in a range of 30.43–89.85%, with mean group values of 55.82% and 63.8% with aescin used as a control. However, interestingly, the fraction showed no activity against hTYR.

There is a lack of studies in the literature on the activity of natural and synthetic compounds against human hyaluronidases and tyrosinases isolated directly from the blood. To the best of our knowledge, information about the activity of *E. divaricatus* roots against these enzymes was obtained for the first time in this study. In our previous studies, we proved the activity of 75% methanolic *E. divaricatus* fruit extract against hHYAL (76.46–86.13%) [35]. In another study, we evaluated the activity of the *intractum of E. senticosus* fruits. The *intractum* significantly inhibited human hyaluronidase activity in ranges of 58.80–76.32% and 20.00–47.37% with aescin used as a control. The results mean that the *intractum* inhibited hyaluronidase activity with mean group values of 60% and 40% with aescin used as a control [38].

### 2.3. Antioxidant Panel

Free radicals are responsible for many of pathogenic processes in the human body, resulting in the development of, e.g., inflammation-related diseases. Taking into consideration the ability to inhibit hyaluronidase (proinflammatory agent), it is important to evaluate the antioxidative activity of fractions. When evaluated for their reactivity towards the ABTS cation radical, the ethyl acetate and n-butanol fractions exhibited the most significant activity, with values of 9.69 ± 0.0035 µg/mL and 10.10 ± 0.21 µg/mL, respectively. Similarly, against the DPPH radical, these fractions demonstrated the highest potency, with values of 36.83 ± 2.43 µg/mL and 61.49 ± 1.87 µg/mL, respectively. The results are presented in Table 8.

There are few studies on the antioxidant properties of *E. divaricatus* roots. The activity of 75% methanolic extract of *E. divaricatus* roots (0.8 mg/mL) against the DPPH radical after 90 min was 23.00 ± 0.79% [31]. In another study, IC_50_ values of the chloroform and ethanol extracts of *E. divaricatus* roots against the DPPH radical were 50.1 ± 0.5 and 1.2 ± 0.2, respectively. For chelation, IC_50_ values for chloroform and ethanol extracts were 0.9 ± 0.51 mg/mL and 0.8 ± 0.01 mg/mL, respectively [30]. In our previous investigations of *E. divaricatus* fruits, 75% methanolic extract showed moderate activity against the ABTS radical, DPPH, and chelating properties, with IC_50_ values of 280, 1300, and 1450 µg/mL, respectively [35]. In a study by Yu et al., the most active fractions against the DPPH radical were EtOAc and BuOH methanolic extract of *E. senticosus* root. The antioxidant activities in the EtOAc and BuOH fractions were higher than or similar to those of α-tocopherol [39]. Furthermore, concentrated powder from *E. senticosus* produced by Sheng Chang Pharmaceutical inhibited DPPH generation by 58.3 ± 2.8% at 1000 µg/mL [40]. The IC_50_ values for hydrophobic–hydrophilic naringenin-enriched extract (1 mg/mL) against the DPPH radical and ABTS were 138.17 ± 4.28 μg/mL and 18.10 ± 0.20 μg/mL, respectively. The chelating capacity at a concentration of 1 mg/mL was 26.34 ± 1.14% [32]. In turn, the IC_50_ values obtained for essential oil from *E. simonii* leaves were 1125 µg/mL, 945 µg/mL, and 862 µg/mL using the DPPH radical, and the ABTS and FRAP methods, respectively [41]. 

### 2.4. Principal Component Analysis (PCA)

**Figure 3:**
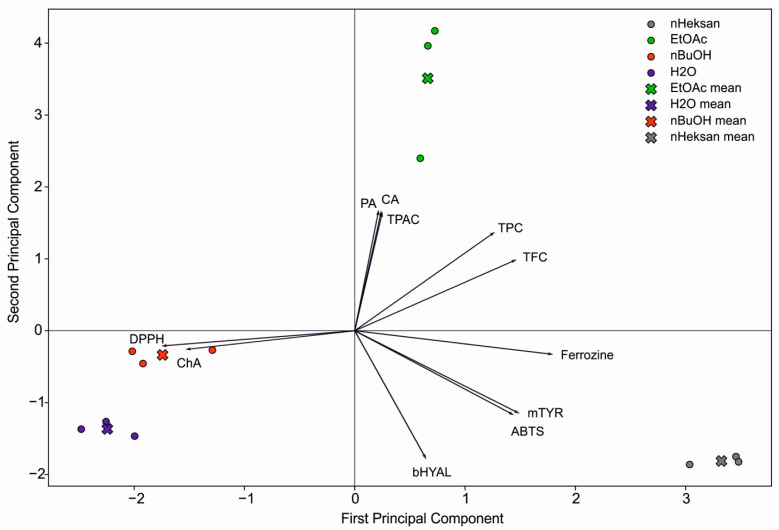
Principal component analysis of the studied fractions of *E. divaricatus* extract based on phenolic compound content and anti-enzymatic and antioxidant activities.

The PCA plot shows (Figure 3) a clear grouping of *E. divaricatus* extract fractions according to the solvents used (n-hexane, ethyl acetate, n-butanol, and water), suggesting that the solvents influence the chemical composition of the fractions, as well as their anti-enzymatic and antioxidant properties. The first principal component explains 44.85% of the variance, and the second principal component explains 41.40%. The H_2_O and n-BuOH fractions, located on the left side of the diagram, are associated with higher chlorogenic acid contents and higher IC_50_ values against DPPH. The n-hexane fraction, located on the right side of the plot, is mainly associated with higher IC_50_ values against ABTS and tyrosinase compared to the other extracts. The EtOAc fraction shows correlations with the vectors for protocatechuic and caffeic acids, as well as TPAC content.

### 2.5. Cytotoxicity Panel

#### Ethyl Acetate Fraction of *E. divaricatus* Does Not Affect the Viability of Normal and Cancerous Skin Cells

**Figure 4:**
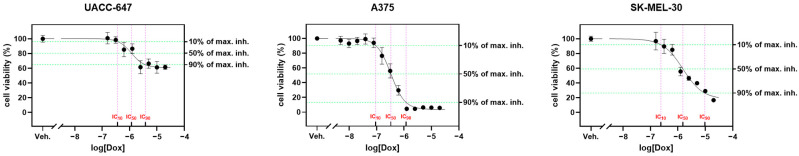
Effect of doxorubicin (DOX) on the viability of UACC-647, A375, and SK-MEL-30 melanoma cell lines. The 10, 50, and 90% levels of maximal viability inhibition are indicated by horizontal dotted lines. The IC_10_, IC_50_, and IC_90_ values are indicated by vertical dotted lines.

Three different human melanoma cell lines, namely SK-MEL-30, UACC-647, and A375, as well as normal BJ fibroblasts, were chosen to investigate the cytotoxicity of the ethyl acetate fraction. We observed no major changes in the viability of the tested cell lines in doses of up to 200 µg/mL, which suggests that the fraction does not have any toxic effects on melanoma cells. Additionally, the fraction did not cause any changes in normal fibroblasts, which may indicate that at the doses used in this study, this fraction is safe. Doxorubicin was used as a control, for which the IC_10_, IC_50_, and IC_90_ values were established (Figure 4). In SK-MEL-30 cells, 10% of maximal inhibition was generated by DOX at a dose of 0.24 µM, 50% of maximal inhibition was generated at a dose of 1.52 µM, and 90% of maximal inhibition was generated at a dose of 9.63 µM. In UACC-647 cells, DOX yielded IC_10_, IC_50_, and IC_90_ values of 0.36, 1.16, and 3.76 µM, respectively. In the A375 cell line, the IC_10_ for DOX was found to be 0.09 µM, the IC_50_ was found to be 0.33 µM, and the IC_90_ was found to be 1.19 µM. The cell lines exhibited differences not only in IC_50_ values but also in the level of inhibition. For instance, the maximal level of inhibition was estimated to be 96.38% in A375 cells. Under the same conditions, the viability of SK-MEL-30 was observed to drop by a maximum of 81.87%. In contrast, the viability of UACC-647 cells was found to be suppressed by only 39.15%.

**Figure 5:**
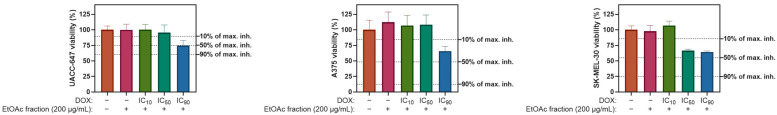
Effect of doxorubicin (DOX) and the EtOAc fraction on the viability of UACC-647, A375, and SK-MEL-30 melanoma cell lines. The cells were exposed to either the IC_10_, IC_50_, or IC_90_ of DOX in combination with the EtOAc fraction at a dose of 200 µg/mL. The 10, 50, and 90% levels of maximal viability inhibition elicited by DOX alone are indicated by horizontal dotted lines.

Natural compounds are frequently used concurrently with chemotherapeutic drugs in the treatment of different diseases. Thus, it is crucial to investigate potential interactions between these types of compounds. In the next step, a potential interaction between DOX and the ethyl acetate fraction was examined (Figure 5). Three melanoma cell lines—UACC-647, A375, and SK-MEL-30—were treated with DOX at three different doses (IC_10_, IC_50_, and IC_90_, as determined independently for each cell line) simultaneously with the EtOAc fraction (200 µg/mL). In all cell lines, a protective effect of the EtOAc fraction was observed, which was concomitant with a decrease in DOX-dependent cytotoxicity. For instance, A375 cells subjected to the EtOAc fraction in combination with the IC_90_ of DOX experienced only a 37% drop in viability (Figure 5, middle panel), whereas DOX alone (at IC_90_) inhibited the viability of A375 cells by 96%.

The literature does not provide sufficient information about the cytotoxicity of the root of *E. divaricatus*. Among the five studied species, *E. henryi* extract showed the strongest inhibition of HL-60 cell line growth, with an IC_50_ value of 270 μg/mL, while for *E. divaricatus*, the IC_50_ value was 650 μg/mL [31]. Fruit extract and hydrophobic–hydrophilic extract from the root of *E. senticosus*, which is enriched with naringenin, did not affect the viability of two cancer cell lines, namely FaDu and HepG2 [32]. The 70% ethanol extract of *E. sessiliflorus* leaves, which is rich in flavonoids, terpenoids, and xylogens, showed weak cytotoxic activity against the A549 cell line [42]. In contrast, taiwanin E, a compound isolated from a branch of *E. trifoliatus*, demonstrated strong cytotoxicity. It exhibited potent antiproliferative effects on the growth of the MCF-7 human breast adenocarcinoma cell line, with an IC_50_ value of 1.47 μM [43]. Elesesterpenes A–K, extracted from the leaves of *E. sessiliflorus* have shown significant antiproliferative activity against several human cancer cell lines, including hepatocellular carcinoma (HepG2), lung adenocarcinoma (A549), and glioblastoma multiforme (LN229), with IC_50_ values ranging from 1.05 to 8.60 μM [44].

## 3. Materials and Methods

### 3.1. Chemicals and Reagents

The standards of eleutheroside B ≥ 98.0% (HPLC) and eleutheroside E ≥ 98.0% (HPLC), protocatechuic acid ≥ 97%, *p*-hydroxybenzoic acid 99%, vanillic acid ≥ 97%, caffeic acid ≥ 98%, ferulic acid ≥ 99%, rosmarinic acid ≥ 98%, DMEM, RPMI 1649 Medium, phosphate buffered saline (PBS), gradient grade acetonitrile, and trifluoroacetic acid ≥ 99%, ascorbic acid, 2(3)-t-butylhydroquinone monomethyl ether (BHA), 2(3)-t-butyl-4-hydroxyanisole, hyaluronic acid (IV), aescin > 95%, hyaluronidase from bovine testes, hexadecyltrimethylammonium bromide (CTAB), L-tyrosine ≥ 98%, kojic acid, and muschroom’s tyrosinase were obtained from Sigma-Aldrich (St. Louis, MO, USA). Additionally, 2,2-diphenyl-1-picrylhydrazyl (DPPH), 2,2′-azinobis-(3-ethylbenzthiazoline-6-sulfonic acid) (ABTS), potassium persulfate, 3-(2-Pyridyl)-5,6-diphenyl-1,2,4-triazine-p,p′-disulfonic acid monosodium salt hydrate 97% (ferrozine), iron(II) chloride tetrahydrate >98% (FeCl_2_ × 4H_2_O), 1,3,5-Tri(2-pyridyl)-2,4,6-triazine (TPTZ), iron (III) chloride > 98% (FeCl_3_), aluminum chloride (AlCl_3_), potassium acetate, Folin−Ciocalteu reagent, sodium nitrite, sodium molybdate, and 6-hydroxy-2,5,7,8-tetramethylchroman-2-carboxylic acid (Trolox) were used. All these substances were purchased from Sigma-Aldrich Corp (Saint Louis, MO, USA). The solvents used for extraction were sourced from Avantor Performance Materials (Gliwice, Poland).

### 3.2. Extraction and Plant Material

The roots of *E. divaricatus* were harvested from the Arboretum of the Warsaw University of Life Sciences in Rogów, Poland, in October 2020. The identification of the raw material was carried out by prof. Daniel Załuski. The extraction process began by preparing an extract from 5 g of *E. divaricatus* root. The root was dried for 4 weeks at room temperature in a dark place. Then, 50 mL of chloroform was poured over the ground root and left for 24 h. Next, the mixture was placed in an ultrasonic bath for 15 min, and the resulting solution was filtered. This process was repeated three times. The filtrate was then concentrated using a vacuum evaporator at 40 °C. After the initial chloroform extraction, the raw material underwent subsequent extractions with ethyl acetate and 75% methanol. The extraction procedure for these solvents was identical to that used for chloroform.

Next, the extracts were tested against hyaluronidase and tyrosinase, and the most active one was subjected to fractionation using liquid–liquid extraction. A 75% methanolic extract of *E. divaricatus* was prepared in a larger amount. Then, 115.109 g of root powder was extracted with 75% methanol solution for three days at room temperature. The flask was then placed in an ultrasonic bath for 15 min. The mixture was filtered, and the crude was poured over 50 mL of 75% MeOH and placed back in a water bath. The procedure was repeated until the filtrate was decolorized.

The obtained extract was concentrated in a vacuum evaporator at 40 °C, yielding 190.08 g/kg of raw material. This residue was fractionated using liquid–liquid extraction (Figure 1).

#### Liquid–Liquid Extraction of Polyphenols

**Figure 6:**
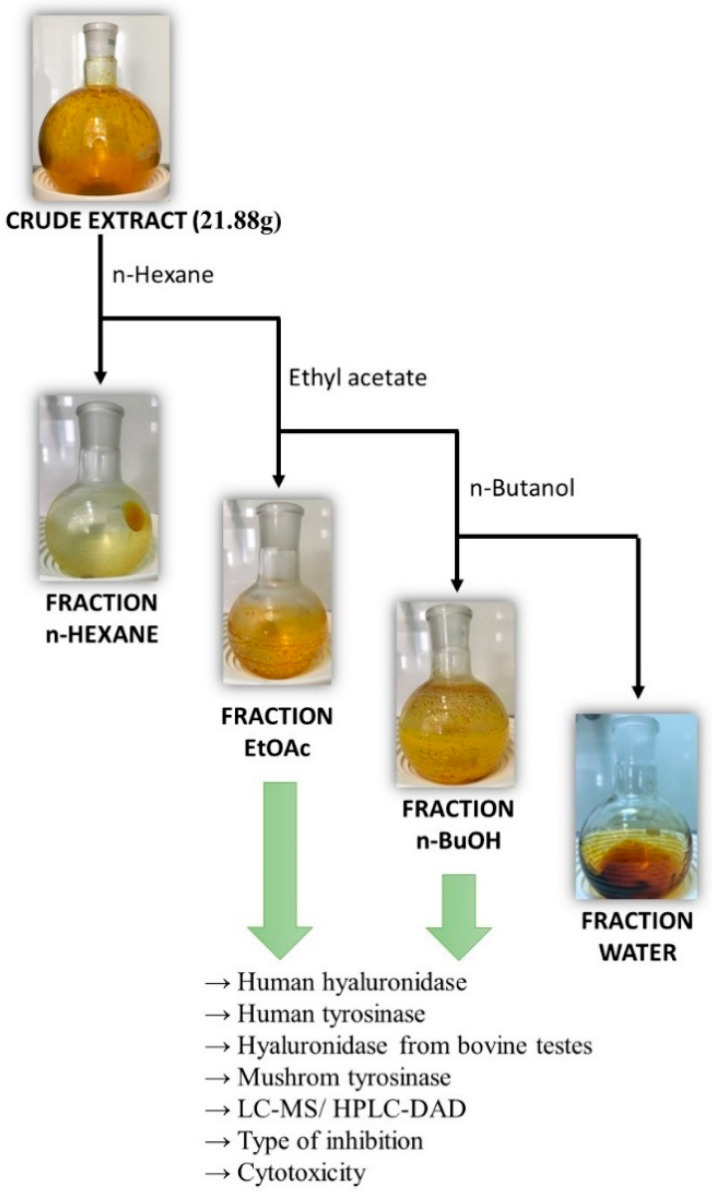
Scheme of liquid–liquid extraction of the 75% methanol extract.

The dried 75% methanol extract, obtained as described in Section 2.2, was suspended in 250 mL of water and extracted with 100 mL of n-hexane. The organic layer was collected, and the procedure was repeated twice using n-hexane. The aqueous solution was then extracted with 100 mL of ethyl acetate and, finally, with 100 mL of saturated n-butanol, as shown in Figure 6. The obtained fractions were concentrated in a vacuum evaporator at 40 °C. The obtained fractions were stored in a refrigerator.

### 3.3. Phytochemical Panel 

#### 3.3.1. Chemical Composition 

##### Determination of Total Phenolic Content (TPC)

A modified Folin–Ciocalteu method was employed to quantify the total phenolic content [45]. First, 25 μL of extract (1 mg/mL in methanol) was combined with 25 μL the Folin−Ciocalteu reagent (diluted 1:3 in pure water) in a 1:1 ratio. After adding 150 μL of distilled water, the mixture was incubated for 5 min. A solution of sodium carbonate (10%) was then added, and the mixture was left to incubate in the dark at room temperature for an hour. The absorbance of the solution was measured at a wavelength of 750 nm. The total phenolic content (TPC) results are expressed in terms of milligrams of gallic acid (GA) equivalents (GAE) per gram of the sample (mg GAE/g sample).

##### Determination of Total Flavonoid Content (TFC)

The total flavonoid content was quantified using a method that relies on the reaction between aluminum chloride (AlCl_3_) and flavonoids [46]. Briefly, 25 μL extract (1 mg/mL in methanol) was mixed with 75 μL ethanol. Subsequently, 10% aluminum chloride (10 μL) and 1M potassium acetate (10 μL) were added to the mixture. After adding 130 μL of distilled water, the mixture was incubated for 30 min. The absorbance of the solution was then measured at a wavelength of 510 nm. The total flavonoid content (TFC) results are expressed as milligrams of quercetin equivalents (QE) per gram of the sample (mg QE/g sample).

##### Determination of Total Phenolic Acid Content (TPAC) 

The total phenolic acid content was determined following the method outlined in the Polish Pharmacopeia VI [47]. First, 25 μL of extract (1 mg/mL in methanol) was combined with 150 distilled water (150 μL), hydrochloric acid (25 μL, 0.5M), and Arnov’s reagent (25 μL, a solution of 10.0 g of sodium molybdate and 10.0 g of sodium nitrite in 100 mL of distilled water). Then, 25 μL of solution of sodium hydroxide (1M) was added to the mixture, which was immediately measured at a wavelength of 492 nm. The total phenolic content (TPC) results are expressed in terms of milligrams of caffeic acid (CA) equivalents (CAE) per gram of the sample (mg CAE/g sample).

#### 3.3.2. Chromatographic Analysis

All standards, formic acid, and MS-grade acetonitrile were from Sigma-Aldrich (St. Louis, MO, USA). MS data were acquired using an Infnity Series II ultra-high-performance liquid chromatograph (UHPLC) with an Agilent 6224 ESI/TOF mass detector (Agilent Technologies, Santa Clara, CA, USA). The conditions were as follows: an RP18 reversed-phase Titan column (Supelco, Sigma-Aldrich, Burlington, MA, USA) (10 cm × 2.1 mm i.d., 1.9 µm particle size), a thermostat temperature of 30 °C, and a flow rate of 0.2 mL/min. Water with 0.05% formic acid (solvent A) and acetonitrile with 0.05% formic acid (solvent B) were used as the mobile phase composition. The gradient elution program was as follows: 0–8 min from 97% A to 95% A, 8–15 min at 95% A, 15–29 min from 95% A to 85% A, 29–40 min at 85% A, 40–50 min from 85% A to 80% A, and 50–60 min from 80% A to 65%. LC–MS conditions were as follows: drying gas temperature of 325 °C, drying gas flow of 8 L min^−1^, nebulizer pressure of 30 psi, capillary voltage of 3500 V, and a 65 V skimmer. The voltage of the fragmentator was 220 V. Ions were acquired in the range of 100 to 1200 *m*/*z* in negative ions. 

Quantitative analyses were performed using an EliteLaChrom chromatograph equipped with a PDA detector and EZChrom Elite software (Version 3.3.2 SP2 build 3.3.2.1037) from Merck (Darmstadt, Germany) and an RP18 reversed-phase Kinetex column measuring 25 cm × 4.6 mm i.d. with a particle size of 5 μm (Phenomenex, Torrance, CA, USA). The elution conditions are described above. The flow rate was set at 1.0mL/min, and data were collected within the wavelength range of 190 to 400 nm. The identities of the compounds were confirmed by comparing their retention times and UV spectra with those of corresponding standards. Quantitative analysis was carried out at the following specific wavelengths for each compound: 260 nm for protocatechuic acid and 325 nm for chlorogenic acid and dicaffeoylquinic acid.

### 3.4. Enzymatic Panel 

#### 3.4.1. Bovine Hyaluronidase Inhibition Assay

Bovine hyaluronidase inhibitor assays were conducted in 96-well plates, following a modified method originally described by Di Ferrante [48] and Studzińska-Sroka [49]. The activity of the compounds or extracts was determined based on the precipitation of non-hydrolyzed hyaluronic acid using cetyltrimethylammonium bromide (CTAB). A 10% water solution of DMSO was used to dissolve the extract. Then, 15 μL of extracts at concentrations of 10.0, 1.0, and 0.1 mg/mL in the well were mixed with an acetate buffer (15 μL, pH = 5.35), an incubation buffer (25 μL, pH = 5.35, 0.01% BSA, 0.45% NaCl), and an enzyme solution (25 μL, 30 U/mL in the incubation buffer). This mixture was incubated at 37 °C for 10 min. Following this, a hyaluronic acid solution (25 μL, 0.3 mg/mL in an acetate buffer with a pH of 5.35) was added. The plates were then incubated for an additional 45 min at 37 °C. After incubation, non-hydrolyzed hyaluronic acid was precipitated by adding 2.5% CTAB (200 μL). The plates were then maintained at 25 °C for 10 min. The intensity of the complex formation was measured at a wavelength of 600 nm. The presence of inhibition was determined by measuring the absorbance of the solution without the inhibitor (A_C_) and the enzyme (A_T_). All samples were tested in triplicate. The inhibition of hyaluronidase was calculated using a specific equation, with aescin used as a standard.

$${INH}_{HYAL}=\frac{A_{S}-A_{C}}{A_{T}-A_{C}}*100\%$$

A_S_—absorbance of the HA + sample + enzyme; 

A_C_—absorbance of the HA + enzyme; 

A_T_—absorbance of the HA + sample. 

#### 3.4.2. Human Serum Hyaluronidase from Children Diagnosed with Acute Lymphoblastic Leukemia 

##### Blood Samples

Five boys (3, 4, 4, 5, and 17 years old) diagnosed with acute lymphoblastic leukemia (ALL) before starting treatment were included in the study. These patients were diagnosed at the Department of Pediatric Hematology and Oncology at Jurasz University Hospital in Bydgoszcz, Poland, between 2019 and 2020. Venous blood samples were collected from each child while fasting and placed into serum tubes supplied by Becton Dickinson, located in Franklin Lakes, NJ, USA. The blood samples were left to clot at room temperature for 30 min, then centrifuged for 20 min at 2000× *g*, also at room temperature. The samples were then collected and stored at a temperature of −80 °C until the time of analysis. The study was approved by the local Bioethics Committee (approval number 608/2019) and was conducted in accordance with the Declaration of Helsinki.

##### Level of Human Serum Hyaluronidase 

A commercially available kit, the LS-F6310 Human Hyaluronidase (Sandwich ELISA) ELISA Kit (LSBio, Lynnwood, WA, USA), was used to measure the concentration of human hyaluronidase in serum. This kit functions based on the Sandwich assay principle and can detect hyaluronidase levels down to a certain limit (0.115 nanograms per milliliter).

##### Human Serum Hyaluronidase Inhibition by the Ethyl Acetate Fraction 

The inhibition of human serum hyaluronidase was assessed using modified methods [50]. The activity of the compounds or extracts was determined by precipitating non-hydrolyzed hyaluronic acid with cetyltrimethylammonium bromide (CTAB). In brief, 10 μL of ethyl acetate fraction (1 mg/mL) and 50 μL of serum were incubated at 37 °C for 15 min. Following this, a solution of hyaluronic acid (0.3 mg/mL in an acetate buffer with a pH of 5.35) was added in a volume of 40 μL. The plates were then incubated for an additional 45 min at 37 °C. After incubation, non-hydrolyzed hyaluronic acid was precipitated by adding 2.5% CTAB. The plates were then shaken at 25 °C for 10 min. The intensity of the complex formation was measured at a wavelength of 600 nm. All samples were tested in triplicate. The inhibition of hyaluronidase was calculated using a specific equation, with aescin used as a standard.

$${INH}_{HYAL}=\frac{A_{S}-A_{C}}{A_{T}-A_{C}}*100\%$$

A_S_—absorbance of the HA + sample + enzyme; 

A_C_—absorbance of the HA + enzyme;

A_T_—absorbance of the HA + sample. 

#### 3.4.3. Tyrosinase Inhibition Assay

Tyrosinase inhibitor assays were conducted in 96-well plates using a modified method originally described by Wróbel-Biedrawa [51]. The process involves the conversion of L-tyrosinase to L-DOPA, then to DOPA-quinone, facilitated by the tyrosinase enzyme. This reaction results in the solution turning brown. In brief, 10 μL of the sample (concentrations of 10.0, 1.0, and 0.1 mg/mL in 10% DMSO) was combined with 150 μL of a phosphoric buffer containing mushroom tyrosinase (pH = 6.88, 100 U/mL). This mixture was then incubated for 10 min at room temperature. A control (A_C_) was also prepared that did not contain any inhibitor. Following incubation, L-tyrosine (0.3 mg/mL) was added to each well, and the absorbance was measured at 492 nm (using a kinetic model every 5 min). Two time points (t_1_ and t_2_) were selected within the linear range of the graph. All samples were tested in triplicate. The inhibition of tyrosinase was calculated using a specific equation, with kojic acid used as a standard.

$${INH}_{TYR}=\frac{A_{C}-A_{S}}{A_{C}}*100\%$$

A_S_—the difference in absorbance between times t_2_ and t_1_ for the sample; 

A_C_—the difference in absorbance between times t_2_ and t_1_ for the positive control. 

### 3.5. Antioxidant Panel 

#### 3.5.1. ABTS Free Radical Scavenging Activity 

The ABTS free radical scavenging test was conducted following the method outlined by Wu et al. [52] A working solution of ABTS+ was prepared by combining 10 mL of ABTS (7 mM in H_2_O) with 10 mL of potassium persulfate (2.45 mM in H_2_O). This mixture was then left to incubate in the dark for 12 h. The ABTS solution was subsequently diluted with water until it reached an absorbance of 0.700 ± 0.03 at 405 nm. Then, volumes of 10 μL of extracts at concentrations of 1 mg/mL, 0.1 mg/mL, and 0.01 mg/mL (dissolved in MeOH) were combined with 190 μL of the ABTS+ solution and incubated for 30 min. The absorbance at 405 nm was measured after the incubation period. Butylated hydroxyanisole (BHA) was used as a control. The antioxidant activity was then calculated using a specific equation.

$${INH}_{ABTS}=\frac{A_{C}-A_{S}}{A_{C}}*100\%$$

A_S_—the absorbance for sample + ABTS; 

A_C—_the absorbance without sample + ABTS.

#### 3.5.2. DPPH Free Radical Scavenging Activity 

The DPPH free radical scavenging test was conducted according to the procedure established by Naseer et al. [53]. A working solution of DPPH• was prepared by dissolving 24 mg of DPPH in 100 mL of distilled water. This solution was further diluted with methanol until it reached an absorbance of 0.900 ± 0.03 at 515 nm. Subsequently, volumes of 10 μL of extracts at concentrations of 1 mg/mL, 0.1 mg/mL, and 0.01 mg/mL (dissolved in MeOH) were combined with 190 μL of the DPPH• solution and allowed to incubate for 60 min. The absorbance at 515 nm was measured post incubation. Butylated hydroxyanisole (BHA) was used as a control. The antioxidant activity was then calculated using a specified equation.

$${INH}_{DPPH}=\frac{A_{C}-A_{S}}{A_{C}}*100\%$$

A_S_—the absorbance for sample + DPPH;

A_C_—the absorbance without sample + DPPH.

#### 3.5.3. Iron (II) Ion Chelation Assay 

The ion chelation assay was conducted using the method proposed by Li et al. [54]. Initially, 100 μL of extract at a concentration of 1.0 mg/mL was combined with methanol (150 μL, MeOH) and iron(II) chloride (5 μL, FeCl_2_) at a concentration of 2 mM. Subsequently, 5 μL of ferrozine was added at a concentration of 5 mM. Following a period of incubation, the absorbance was measured at a wavelength of 510 nm. The degree of chelation was then calculated using a specific equation, with ethylenediaminetetraacetic acid (EDTA) serving as a positive control.

$${INH}_{{Fe}^{2+}}=\frac{A_{C}-A_{S}}{A_{C}}*100\%$$

A_S_—the absorbance for sample + ferrozine + FeCl_2_;

A_C_—the absorbance without sample + ferrozine + FeCl_2_.

### 3.6. Cytotoxicity Panel

#### Cell Culture and Cytotoxicity Assessment

The following human melanoma cell lines were used in this study: A375 (ATCC:CRL-1619), UACC-647 (CVCL_4049), and SK-MEL-30 (DSMZ:ACC 151). A375 cells were maintained in Dulbecco’s Modified Eagle’s Medium (DMEM). UACC-647 and SK-MEL-30 were cultured in RPMI-1640. All basal media were supplemented with fetal bovine serum (FBS) at a final concentration of 10%. The control used in the study was BJ foreskin fibroblasts (ATCC:CRL-2522). To facilitate the growth of BJ cells, Eagle’s Minimum Essential Medium (EMEM) with 10% FBS was utilized. Tissue culture-treated Petri dishes were used to support all cell lines. A temperature of 37 °C and 5% CO_2_ were maintained routinely. The cells were expanded for a few passages, then seeded onto 96-well plates, where they were left to attach overnight. The following day, the cells were exposed to *E. divaricatus* fractionated extracts at descending concentrations, starting from 200 µg/mL, or to a vehicle (DMSO, 0.1%). Alternatively, the cells were treated with doxorubicin (DOX) to independently determine the inhibitory concentrations causing the 10, 50, and 90% levels of maximal inhibition (IC_10_, IC_50_, and IC_90_, respectively) of cellular viability for each melanoma cell line. Then, the melanoma cells were treated with the DOX at IC_10_, IC_50_, or IC_90_, followed by the addition of the fractions of the extracts from *E. divaricatus* at a dose of 200 µg/mL. Upon 24 h of incubation, the viability of the cells was assessed by MTT assay.

### 3.7. Statistics

Dose–response curves were obtained using GraphPad Prism 8.4.3 software running on a personal computer. Experimental datapoints were fitted to the sigmoidal equation, and the IC_50_ value was calculated. IC_10_ and IC_90_ values were computed using a web tool available at https://www.graphpad.com/ accessed on 20 May 2023.

Statistically significant differences were assessed using Friedman’s ANOVA. The Dunn–Bonferroni–Holm test was utilized as a post hoc test. Principal component analysis (PCA) was employed to demonstrate differences between the studied fractions. Analyses were conducted in PQStat software 1.8.6.

## 4. Conclusions

Advances in phytochemical analysis and phytopharmacology make it possible to identify biologically active substances and to assess their mechanisms of molecular activity. Most knowledge about plant-based compounds comes from ethnopharmacology, in combination with modern concepts in that field, allowing for the development of effective, safe, and standardized plant extracts.

The obtained results confirm the effectiveness of using *E. divaricatus* in TCM to treat inflammation- and immune-related diseases. Simultaneously, in light of what has already been published we should be careful when this plant is administered together with chemotherapy, which is a popular approach to strengthen the body. From this study, it is clear that the EtAOc fraction may decrease the activity of doxorubicin, while it does not inhibit human tyrosinase isolated from the leukemic children. Therefore, it is questionable whether adaptogenic or/and immunostimulant compounds should be use during chemotherapy. Keeping these questions in mind, further research using in vivo models is needed.

## Figures and Tables

**Table 1:** Chemical composition of the 75% methanol extract of *E. divaricatus* after its fractionation using liquid–liquid extraction [mg/g ext. ± SD] and mass of fraction [g]. Different superscript lowercase letters indicate a statistically significant difference between the fractions within the same column, with *p* < 0.05.

Fraction	TPC [mgGAE/g]	TFC [mgQE/g]	TPAC [mgCAE/g]	Mass of Fraction [g]
n-Hexane	68.16 ± 1.32 ^ab^	21.80 ± 1.53 ^ab^	1.03 ± 0.18 ^ab^	2.02
Ethyl acetate (EtOAc)	110.89 ± 6.32 ^b^	27.95 ± 4.11 ^b^	2.81 ± 0.48 ^b^	1.02
n-Butanol (n-BuOH)	22.03 ± 0.77 ^ab^	0.65 ± 0.77 ^a^	1.74 ± 0.24 ^ab^	5.11
Water	4.02 ± 2.88 ^a^	3.36 ± 2.17 ^ab^	0.55 ± 0.033 ^a^	9.26

**Table 2:** Phenolic composition of *E. divaricatus* fractions obtained using UHPLC-DAD/ESI-TOF-MS.

N^o^	Rt (min)	Observed Ion Mass [M − H]^−^/(Fragments)	Δ ppm	Formula	Identified
1	5.57	153.01973	2.58	C7H6O4	Protocatechuic acid *
2	8.73	137.02481	2.84	C7H6O3	Hydroxybenzoic acid
3	14.00	289.07203	0.92	C15H14O6	Catechin *
4	15.20	179.03505 (135, 191)	0.38	C9H8O4	Caffeic acid *
5	16.42	353.08835 (191, 179)	1.54	C16H18O9	Chlorogenic acid *
6	26.16	389.12423 (227)	0.10	C20H22O8	Piceid (Resveratrol der.)
7	36.84	515.12021 (353)	1.38	C25H24O12	3,5-dicaffeoylquinic acid *
8	37.63	515.12048 (353)	1.90	C25H24O12	Dicaffeoylquinic acid
9	50.90	515.12035 (353)	1.65	C25H24O12	4,5-dicaffeoylquinic acid *
10	59.33	577.13521 (198, 385)	0.10	C30H26O12	Diferulic acid derivative

*—identification was confirmed by comparison with standards.

**Table 3:** The results of the quantification of the main identified components, expressed in mg per g of dried fractions.

	PA	CA	ChA	3,5-DCA	DCA	4,5-DCA
n-Hexane	0.017 ± 0.001	0.077 ± 0.006	0.593 ± 0.020	0.283 ± 0.001	0.334 ± 0.001	0.150 ± 0.009
Ethyl acetate	9.293 ± 0.105	24.018 ± 0.045	16.653 ± 0.055	126.97 ± 3.08	150.63 ± 3.65	26.615 ± 0.253
n-Butanol	0.492 ± 0.001	0.595 ± 0.003	59.198 ± 0.153	24.87 ± 0.109	29.51 ± 0.129	6.383 ± 0.061
Water	0.169 ± 0.008	0.281 ± 0.004	44.360 ± 0.102	1.294 ± 0.011	1.540 ± 0.013	0.129 ± 0.009

PA—protocatechuic acid; CA—caffeic acid; ChA—chlorogenic acid; DCA—dicaffeoylquinic acid.

**Table 4:** Activity of polar and nonpolar extracts against hyaluronidase from bovine testes and mushroom tyrosinase. IC_50_ values are shown in µg/mL. Different superscript lowercase letters indicate a statistically significant difference between the fractions within the same column, with *p* < 0.05.

Type of Extract	bHYAL	mTYR
Chloroform (CHCl_3_)	111.73 ± 0.75 ^a^	188.50 ± 1.83 ^ab^
Ethyl acetate	104.13 ± 2.51 ^a^	274.37 ± 3.69 ^b^
75% methanol	100.80 ± 0.9 ^a^	103.60 ± 4.23 ^a^
CHCl_3_:MeOH:H_2_O	181.27 ± 0.92 ^a^	221.83 ± 2.21 ^ab^

CHCl_3_:MeOH:H_2_O volume ratios, 7:3:0.4; bHYAL—bovine hyaluronidase; mTYR—mushroom tyrosinase.

**Table 5:** Activity of fractions obtained from methanolic extract of *E. divaricatus* root against bHYAL and mTYR. IC_50_ values are shown in µg/mL. Different superscript lowercase letters indicate a statistically significant difference between the fractions themselves and between the fractions and control within the same column, with *p* < 0.05.

Type of Fraction	bHYAL	mTYR
n-Hexane	94.44 ± 0.80 ^ab^	207.50 ± 3.63 ^b^
Ethyl acetate	27.50 ± 0.65 ^a^	65.50 ± 1.35 ^ab^
n-Butanol	56.10 ± 6.86 ^ab^	85.40 ± 2.51 ^ab^
Water	71.60 ± 3.87 ^ab^	81.10 ± 5.32 ^ab^
Aescin	388.8 ± 1.81 ^b^	
Kojic acid		4.44 ± 0.06 ^a^

bHYAL—bovine hyaluronidase; mTYR—mushroom tyrosinase.

**Table 6:** Activity of selective phenolic acid and eleutherosides against bHYAL and mTYR. IC_50_ values are shown in µg/mL. Different superscript lowercase letters indicate a statistically significant difference between the different acids within the same column, with *p* < 0.05.

Standard	bHYAL	mTYR
Eleutheroside B	NA	NA
Eleutheroside E	NA	NA
Eleutheroside E_1_	NA	NA
Caffeic acid	111.34 ± 3.59 ^a^	56.22 ± 0.67 ^a^
Chlorogenic acid	519.14 ± 17.94 ^ab^	107.52 ± 3.46 ^ab^
Protocatechuic acid	920.20 ± 87.71 ^b^	134.57 ± 3.46 ^b^

bHYAL—bovine hyaluronidase; mTYR—mushroom tyrosinase.

**Table 7:** Activity of ethyl acetate fraction (EtOAc) against hyaluronidase and tyrosinase from human serum isolated from the blood children diagnosed with acute lymphoblastic leukemia (N°). Results are presented in %. Different superscript lowercase letters indicate a statistically significant difference between the samples within the same column, with *p* < 0.05.

N°	Level of hHYAL [ng/mL]	hHYAL [%]	Level of hTYR [ng/mL]	hTYR [%]
		EtOAc		EtOAc
		Mean ± SD		Mean ± SD
1	95.27	53.47 ± 12.37 ^ab^	4.68	NA
2	116.90	89.85 ± 7.73 ^b^	9.31	NA
3	162.15	66.67 ± 20.00 ^ab^	15.26	NA
4	81.86	38.71 ± 5.59 ^ab^	78.94	NA
5	25.20	30.43 ± 3.07 ^a^	52.17	NA
Mean value	96.27	55.82	32.07	-

NA—Not Active.

**Table 8:** Antioxidants activity of *Eleutherococcus divaricatus* fractions. IC_50_ values are shown in µg/mL. The results for ferrozine are shown in %. Different superscript lowercase letters indicate a statistically significant difference between the fractions themselves and between the fractions and control within the same column, with *p* < 0.05.

Fraction	IC_50_ ABTS	IC_50_ DPPH	Ferrozine *
n-Hexane	80.20 ± 5.40 ^b^	NA	80.15 ± 0.73
Ethyl acetate	9.69 ± 0.035 ^ab^	36.83 ± 2.43 ^ab^	34.44 ± 3.99
n-Butanol	10.10 ± 0.21 ^ab^	61.49 ± 1.87 ^ab^	23.52 ± 3.24
Water	24.99 ± 0.095 ^ab^	106.10 ± 4.51 ^b^	4.09 ± 2.48
BHA	2.35 ± 0.11 ^a^	62.52 ± 4.13 ^ab^	
AA	2.27 ± 0.07 ^a^	24.93 ± 0.28 ^ab^	
TROLOX	2.85 ± 0.18 ^ab^	13.68 ± 3.53 ^a^	
EDTA			98.9 ± 0.01

NA—not active; * results for 1 mg/mL.

## Data Availability

The data presented in this study are available upon request from the corresponding author.
